# Brain Death and Organ Donation in Romania: A Nationwide Survey of Intensivists’ Perceptions and Clinical Practices

**DOI:** 10.3390/jcm15051769

**Published:** 2026-02-26

**Authors:** Alberto Bacușcă, Grigore Tinică, Alexandru Burlacu, Andrei Țăruș, Domnica Bacușcă, Mihail Enache, Agnes Bacușcă, Bianca Hanganu, Cristina Gavriluță, Beatrice Gabriela Ioan

**Affiliations:** 1 Department of Cardiovascular Surgery, Cardiovascular Diseases Institute, 700503 Iași, Romania; alberto-bacusca@email.umfiasi.ro (A.B.); grigore.tinica@umfiasi.ro (G.T.); andrei-v-tarus@d.umfiasi.ro (A.Ț.); mihail.enache@umfiasi.ro (M.E.); 2 Department of Internal Medicine, “Grigore T. Popa” University of Medicine and Pharmacy, 700115 Iași, Romania; elena-domnica.bacusca@email.umfiasi.ro (D.B.); agnes.bacusca@umfiasi.ro (A.B.); bianca-hanganu@umfiasi.ro (B.H.); beatrice.ioan@umfiasi.ro (B.G.I.); 3 Department of Interventional Cardiology, Cardiovascular Diseases Institute, 700503 Iași, Romania; 4 Institute of Legal Medicine, 700455 Iași, Romania; 5 Department of Sociology and Social Work, Faculty of Philosophy and Social-Political Sciences, “Alexandru Ioan Cuza” University of Iași, 700506 Iași, Romania; cristina_gavriluta@yahoo.fr

**Keywords:** brain death, organ donation, questionnaire-based survey, intensive care physicians, health services research

## Abstract

**Background/Objectives:** A persistent mismatch between organ supply and transplant demand affects healthcare systems worldwide, particularly in underdeveloped and transitional systems. Intensive care units (ICUs) represent the primary setting for donor identification following brain death, placing intensive care physicians at the center of organ donation pathways. This nationwide cross-sectional survey aimed to evaluate Romanian intensivists’ knowledge, attitudes, and reported clinical practices regarding brain death determination, communication with families, and system-level barriers to organ donation, to identify modifiable factors relevant to transplant policy development. **Methods:** A prospective, nationwide, questionnaire-based survey was conducted among intensive care physicians in Romania. The structured questionnaire explored their knowledge and attitudes regarding brain death, communication with families, involvement in donation processes, ethical perceptions, and views on the organization of the transplant system. The survey was distributed through the Romanian Society of Anesthesia and Intensive Care, and descriptive exploratory analyses were performed. **Results:** A total of 117 ICU physicians participated (mean age 41.0 ± 9.9 years). Although 84.6% agreed with the current brain death diagnostic criteria, and 83% considered the protocol sufficiently clear. The mean number of brain-dead patients managed annually was 8.25 ± 12.90. 69.3% of respondents perceived communication competencies as insufficient. 77.8% considered family consent decisive in donation decisions, while 87% supported the establishment of a national donor registry and 77% favored a donor card system. Organ procurement was reported as a priority in only 38.5% of ICUs. Institutional prioritization of organ procurement and structured training was inconsistent. **Conclusions:** This nationwide survey identifies key educational, organizational, and systemic barriers limiting organ donation performance in Romania. Targeted training, improved communication strategies, integration of donation pathways into routine intensive care practice, and the adoption of national consent instruments represent essential clinical and policy priorities for low-performing transplant systems.

## 1. Introduction

A substantial mismatch between organ supply and transplant demand persists across global transplant systems, with a disproportionately greater impact observed in underdeveloped healthcare systems, including those of Eastern European countries such as Romania [1]. Intensivists may contribute to narrowing this gap by implementing systematic strategies for the identification and preservation of potential organ donors in intensive care units (ICUs) following death determined by neurological criteria. When ICU physicians provide care to patients with imminent or established brain death, or when the continuation of active treatment is deemed futile, the option of organ donation should consistently be considered [2]. The principle that donation should represent a routine component of end-of-life care is now recognized in many countries [3].

More than 90% of organ donors are patients who die after the irreversible cessation of all brain functions in Intensive Care Units. The transplantation pathway is initiated in intensive care units with the formal declaration of brain death and the recognition of a patient as a potential donor, positioning ICU physicians as key decision-makers in the organ procurement process and as the primary interface with families during consent discussions [4].

The reasons why a possible donor does not become an actual donor include failure to identify the donor, failure to report the case, management errors with hemodynamic instability or cardiac arrest resulting in organ deterioration [5]. Because most potential organ donors are in ICUs, critical care management guided by the intensive care physicians plays an essential role.

The donor management must ensure physiological homeostasis to maintain the best possible organ function at the time of procurement, including cardiovascular, respiratory, fluid, electrolyte, hormonal, blood, coagulation, and temperature management to maintain normovolemia, hemodynamic stability, and normothermia [6]. Therefore, the level of training and interest among ICU staff is crucial to the functioning of a highly performing donation system.

Despite the recognized importance of intensive care units in donor identification, no nationwide assessment has systematically evaluated Romanian intensivists’ perspectives on brain death determination, communication practices, and system-level barriers within the transplant framework. Given Romania’s comparatively low organ donation rates within the European Union, understanding professional-level constraints is essential for contextualized policy development.

This study addresses the following research question: What clinical, communicational, and system-level factors influence intensivists’ engagement in organ donation pathways within the Romanian healthcare system? Accordingly, the objective of this study was to provide a structured national assessment of intensivists’ perspectives to identify educational and organizational gaps potentially relevant to transplant system improvement.

## 2. Materials and Methods

### 2.1. Study Design

This study was designed as a prospective, nationwide, quantitative survey of intensive care physicians, conducted using a structured questionnaire developed from the specialized literature. The study was approved by the Research Ethics Committee of the University of Medicine and Pharmacy “Grigore T. Popa” of Iasi (no.114/15 October 2021). The survey was designed for exploratory analysis of clinical practice and system-level factors rather than for population-level prevalence estimation.

### 2.2. Questionnaire Description and Measured Variables

The questionnaire consisted of 57 open- or closed-ended questions, with multiple response options, designed to provide participants who were uncertain about the topic or whose views were not yet fully formed with an alternative way to express their own opinions. The questions were edited according to the 14 criteria described by Likert [7] and Edwards [8]. Participants were asked to indicate their degree of agreement with each statement on a 5-point scale, ranging from 5 = always agree; 4 = mostly agree; 3 = neither agree nor disagree; 2 = mostly disagree; and 1 = total disagreement.

The questionnaire was structured in four sections: the first section included 10 questions aimed at outlining ICU physicians’ opinions regarding brain death and organ donation for transplantation. The second section, comprising 16 questions, addressed aspects related to communication with families of brain-dead patients. The third section, consisting of 15 questions, aimed to capture respondents’ opinions regarding the transplant system and the involvement of medical staff in transplant activity. The fourth section included 5 questions aimed at analyzing personal opinions regarding organ transplantation, allowing the participant to be placed in the role of a potential donor or of a relative of a brain death patient.

Sociodemographic and professional data were collected in the initial part of the questionnaire through 11 questions.

Before completing the questionnaire, respondents were informed in writing about the purpose of the study, the method of data collection, the risks and benefits associated with this research, as well as about the confidentiality of respondents’ personal data and how the results would be used. Participation in this study was voluntary. By moving to the first section of the questionnaire, respondents provided informed consent to participate in the study.

### 2.3. Study Population

The questionnaire was distributed online to all ICU physicians working in Romania via Google Forms (https://docs.google.com/forms/d/1qR468rgC_3NlRnIpHKGus2n8sLKsMczAW9N8wZCDUKw/edit?usp=forms_home&ouid=102041092426220251079 (accessed during the period 21 March 2023–22 June 2023)), with the support of the Romanian Society of Anesthesia and Intensive Care (SRATI), during the period 21 March 2023–22 June 2023. Completing the questionnaire took approximately 25 min.

Inclusion criteria were: (1) board-certified intensivists practicing in Romania during the study period and (2) voluntary participation. Exclusion criteria included non-ICU physicians and questionnaires with more than 20% missing responses. This study was designed as an exploratory national survey to identify trends and patterns in ICU physicians’ perspectives, rather than to estimate population-level prevalence. The survey was distributed nationally through the Romanian Society of Anesthesia and Intensive Care (SRATI), which includes registered ICU physicians across Romania. Participation was voluntary, and no random sampling procedure was applied. Therefore, the term ‘nationwide’ refers to geographic coverage rather than probabilistic representativeness [8].

The questionnaire was developed for exploratory research purposes based on the available literature and demonstrated good internal consistency. External validation was not performed, representing a limitation inherent to nationwide survey-based studies.

### 2.4. Data Analysis

Data were analyzed using SPSS 18.0, applying the appropriate statistical functions. Both descriptive and analytical methods were used, with a 95% significance threshold.

The statistical analyses were primarily descriptive and exploratory, aiming to identify trends and associations rather than to estimate population-level prevalence. Inferential analyses were conducted in an exploratory manner to identify potential associations between variables. Given the exploratory design, findings should be interpreted cautiously, particularly given multiple comparisons.

Scale Reliability analysis. The intercorrelation matrix provides an overview of the degree of association among items. The values are useful to demonstrate that there are no item construction problems and that there is no high degree of similarity. Cronbach’s alpha = 0.809 represents an acceptable value compared with the required threshold (0.700) for validating the use of this questionnaire.

Skewness analysis was conducted to assess the appropriateness of the data distribution for the application of parametric statistical procedures. To assess statistically significant differences between groups at the 95% confidence level, quantitative variables were analyzed using tests selected based on their distributional properties. Student’s *t*-test was used to examine differences in mean values between two independent groups and to evaluate the effect of group membership on continuous outcomes. For comparisons involving three or more groups, FANOVA was used to explore overall intergroup variability. At the same time, the Bonferroni post hoc correction was applied to identify specific pairwise differences and control for multiple testing.

Associations between categorical variables were investigated using the Chi-square (χ^2^) test, enabling the identification of dependency patterns between mutually exclusive outcome categories. Comparisons of ordinal variables across multiple groups were performed using the Kruskal–Wallis test, which detects systematic rank-based differences without reliance on normality assumptions.

## 3. Results

### 3.1. Participants

**Figure 1:**
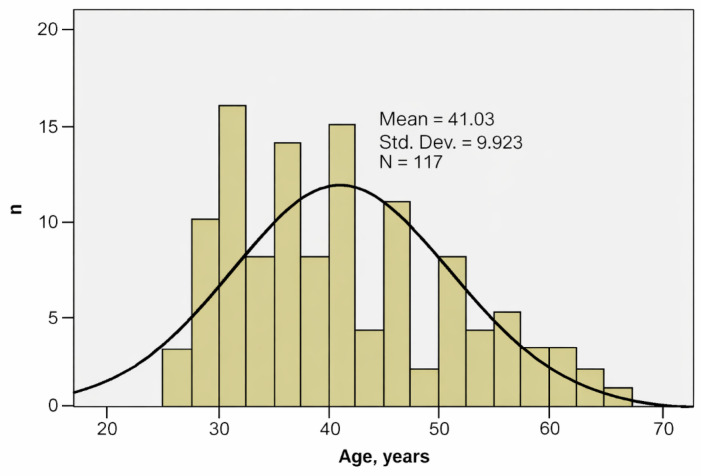
Histogram of participants’ ages (years).

A total of 117 ICU physicians participated in our research, of whom 63.2% were female. The sample represents approximately 6.1% of the total population of ICU physicians registered in the SRATI Statistical Yearbook (*n* = 1909), reflecting national coverage rather than probabilistic representativeness. The age values series was homogeneous, suggesting that statistical significance tests can be applied (Table 1, Figure 1).

Most respondents were married (59.8%), and 10.3% were in a cohabiting relationship; 29.9% were divorced, widowed, or single. The mean professional experience in the ICU was 12.11 years. Of the total sample, 30.8% had studied or practiced abroad, with no significant differences by gender or age. Most frequently, respondents were Christian Orthodox (79.5%); approximately one-third (31.6%) were moderately practicing, while 27.4% were practicing to a large or very large extent.

### 3.2. Opinions Regarding the Concept of Brain Death and Organ Donation

The mean number of brain-dead patients cared for per year is only 8.25 ± 12.90. More than half of respondents stated that ICU physicians know the brain death criteria to a large and very large extent (58.1%). In comparison, nearly one-third of participants rated this aspect as being to a small extent or not at all (29.1%), with no statistically significant differences by age, gender, or marital status. Most respondents agreed with the current brain death criteria (84.6%), and 83% of participants considered that the current protocol for brain death diagnosis is sufficiently clear.

**Figure 2:**
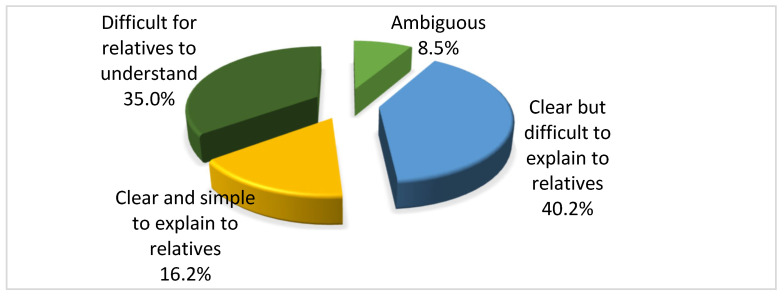
Distribution of the study sample based on viewpoints regarding the concept of brain death.

Over 40% of respondents consider the concept of brain death to be clear but difficult to explain to relatives (40.2%). In comparison, 35% consider it difficult for relatives to understand, with a significant gender difference, i.e., among those who consider the concept of brain death difficult for relatives to understand, 78% were female (*p* = 0.007) (Figure 2).

Fifty percent of respondents state that the process of declaring brain death has a major emotional impact on them; however, the vast majority of physicians are not afraid at all (30.8%) or only to a small extent (65.9%) that applying the brain death diagnostic criteria could lead to false-positive diagnoses of brain death, with most of these being male (52.8%, *p* = 0.043).

Most respondents agree always (40.2%), often (22.3%), and very often (21.4%) that organ donation should be the priority strategy for brain death patients.

A total of 73.5% of participants consider that caring for a brain-dead donor is a stressful experience to a large or very large extent, and 44% consider that expressing empathy toward relatives of brain-dead patients is more complicated than in the case of other patients. Female respondents are more likely to experience stress in this context (70.9%, *p* = 0.011).

### 3.3. Communication with the Family of the Potential Brain-Dead Donors

Our results indicate significant perceived deficiencies in ICU staff’s communication competencies in relation to families of potential brain-dead donors. Thus, 69.3% of respondents did not generally agree with the statement that ICU staff possess genuine communication competencies, while only 30.8% agreed.

At the same time, most participants considered that relatives of brain-dead patients are familiar with this diagnosis only to a small extent (61.5%) or not at all (25.6%). Nevertheless, respondents recognize the decisive role of understanding the concept of brain death in the donation decision: 77.8% consider that it influences the family’s willingness to donate to a large or very large extent.

Only 29.9% of respondents consider that relatives’ presence during the brain death confirmation tests could positively influence their willingness to donate. At the same time, more than half of the participants believe that their presence would have minimal or no impact.

Approximately half of the respondents consider that financially motivating families would not facilitate obtaining their consent for donation. However, 30.8% of participants believe that financial motivation would have a positive impact on consent to donate to a large or very large extent. Nevertheless, 65% of the ICU physicians participating in our study consider that granting a financial reward is not morally justified, irrespective of age, gender, or marital status.

**Figure 3:**
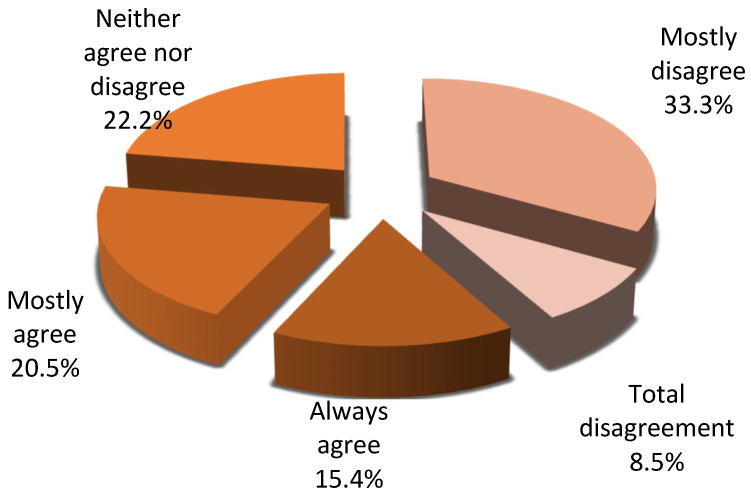
Relatives’ association of the concept of brain death with the patient’s death.

**Figure 4:**
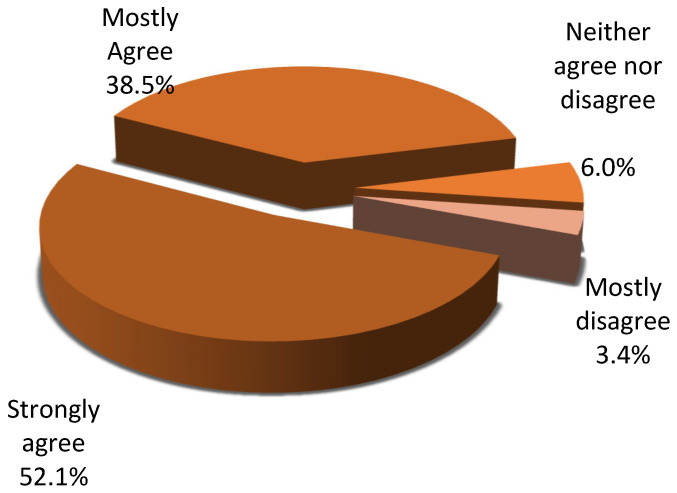
Relatives’ religious beliefs in relation to organ donation.

Almost 40% of respondents consider that families do not understand the importance of organ donation. Furthermore, 8.5% of respondents reported that families do not associate brain death with the patient’s death at all, while 33.3% perceived this association as limited; in contrast, 45.9% indicated that relatives link the two concepts—brain death and donation—to a large or very large extent (Figure 3). Furthermore, 50% of respondents rated the influence of relatives’ religious beliefs on donation decisions as very strong, whereas 38.5% evaluated it as strong. (Figure 4).

In the context of discussions between ICU physicians and families of brain-dead patients, aimed at explaining the patient’s clinical condition, most respondents believe that family members have sufficient time to understand the patient’s medical situation (59%), to reflect on the decision to donate (63.2%), and to consult with other family members (68.4%). By contrast, a considerably smaller proportion of respondents consider that the family has adequate time to regain emotional balance (17.1%). Sixty-three percent of participants believe that discussions with families help them accept reality. In contrast, 52% of participants believe that the family members may perceive these discussions as inappropriate in the given context and may intensify their emotional suffering.

Most respondents (“always”-38.5%, “very often”-19.7%) consider that consent for organ donation belongs to the family when the patient has not previously expressed an option in this regard, while 6.8% express opposing views.

### 3.4. ICU Physicians’ Opinions Regarding the Transplant System and ICU Staff Involvement in Transplant Activity

Participants showed a predominantly reserved opinion regarding Romanian society’s preparedness to implement legislation based on presumed consent, with most considering it prepared to a small extent (46.2%) or not at all (31.6%). At the same time, over 87% of participants consider that a unique national registry of persons who agreed to donate organs and tissues is necessary to a large or very large extent. In addition, 77% of respondents support implementing a donor card–based system as a feasible solution for a clear record of persons wishing to become donors.

Participants consider that the optimal moment for a person to express written consent to organ donation is upon obtaining an identity card (29.1%), upon presenting to a family doctor (23.9%), or upon obtaining a driver’s license (21.4%).

Regarding the risk of abuses related to organ procurement from brain-dead donors, 57.3% of respondents believe this is not an issue in Romania. In comparison, 20.5% believe it exists to a large or very large extent.

A total of 53% of participants consider that ICU medical staff are well prepared, to a large or very large extent, to manage situations in which the family is willing to donate the organs of a brain-dead patient, and over 62% report having the competencies required to preserve the potential donor until donation. Concerning the motivation of medical staff, 63% believe this could increase interest in identifying potential donors; however, only 38% consider financial incentives the most appropriate method, while other motivating factors are mentioned, such as professional development through specialized continuing medical education, broader involvement in procurement-donation activity, psychological counseling, institutional recognition, and reduction in bureaucratic burden.

Organ procurement is a priority in the ICU, where respondents work in only 38.5% of cases, and 44.4% of participants expressed a desire to become more involved in organ donation and transplantation activity. Finally, 73.5% of respondents know who the transplant coordinator/KDP is and how to contact them when identifying a potential donor, while 25.6% state they would be willing to accept the role of KDP or transplant coordinator.

Beyond individual knowledge and attitudes, the present findings suggest that systemic and organizational factors substantially shape clinical practice patterns in organ donation. The fact that organ procurement is reported as a priority in only 38.5% of ICUs indicates heterogeneity at the institutional level. In the absence of formal integration of donation pathways into routine intensive care workflows, donor identification may remain dependent on individual initiative rather than on standardized protocols. This structural variability likely contributes to inconsistent donor conversion rates across institutions.

The reported willingness of 44.4% of respondents to become more involved in donation activity, contrasted with the limited institutional prioritization, suggests a coordination gap between professional motivation and organizational implementation. Unequal access to structured training programs, inconsistent involvement of transplant coordinators, and the absence of clearly defined ICU-level donation triggers may limit the translation of positive attitudes into systematic practice. In low-performing transplant systems, such fragmentation may undermine donor identification despite favorable professional perceptions.

### 3.5. ICU Physicians’ Opinions from the Perspective of Potential Organ Donors or Family Members

From the perspective of personal and family-related decision-making, 66.7% of ICU physicians reported willingness to donate their own organs in the event of being declared brain dead. In comparison, an even higher proportion (70%) indicated that they would consent to organ and tissue donation for a brain-dead family member. These findings suggest a generally favorable attitude toward organ donation among ICU physicians, both at the individual level and when acting as surrogate decision-makers.

## 4. Discussion

This nationwide quantitative survey provides a clinically grounded, system-level assessment of intensive care physicians’ perspectives on brain death and organ donation within a low-performing transplant system, highlighting structural, educational, organizational, and ethical barriers with direct relevance for policy development. Most European countries allocate significant efforts and resources to deepen understanding of the obstacles to organ donation and transplantation, to improve the balance between the number of donated organs and demand. These obstacles are clearly multifactorial, including cultural and religious norms, legal regulations, and the organization of health systems [9,10]. The knowledge and attitudes of medical staff can also significantly impact this equation [11].

In Romania, the transplant system is precarious, with slow development and low performance compared with other European systems. The study was made possible with the support of the Romanian Society of Anesthesia and Intensive Care. Our study, the first of its kind conducted in Romania, aimed to analyze the opinions of ICU physicians regarding organ donation, the concept of brain death, their experience in caring for potential donors, and approaching their families. These aspects have the potential to contribute significantly to the development of a coherent strategy for the Romanian transplantation system.

### 4.1. Opinions Regarding the Concept of Brain Death and Organ Donation

The diagnosis of brain death has significant medical, ethical, and legal consequences, potentially leading to the decision to withdraw life-sustaining measures or to initiate the organ procurement process for transplantation. There is significant variability in brain death diagnostic practices between countries, as well as in clinical decisions made after brain death confirmation [12,13]. In general, brain death determination is performed predominantly in ICUs and is based on clinical examination according to international standards and on additional investigations such as electrophysiological studies, including electroencephalography (EEG), assessment of cerebral blood flow when the patient has received sedatives, has severe facial trauma, or does not tolerate the apnea test, or cerebral angiography, cerebral scintigraphy, and transcranial Doppler [12,14,15].

Strengthening healthcare professionals’ positive attitudes toward brain death could significantly increase donation rates by facilitating the procurement process. Moreover, assuming brain death as a valid determinant of death has a positive effect on healthcare professionals’ comfort level in performing key donation-related tasks [16,17].

Although important progress has been made regarding acceptance of brain death and organ donation, our study shows that a certain level of skepticism persists among ICU physicians regarding the validity of brain death diagnostic criteria, i.e., 15.4% of the physicians surveyed did not agree with the brain death diagnostic criteria. This is also noted by Camut et al., who report that up to 8.3% of professionals dealing with such cases do not consider brain death to be “real”, with the highest proportion of skeptics in the Stroke Unit (36.4%) and the lowest, 2.2%, in the Neurosurgical ICU [18]. This may also relate to the fact that the impact of organ procurement from brain-dead patients could be experienced by healthcare staff as active participation in stopping the patient’s life. Thus, the concept of brain death implies that a patient is considered dead. At the same time, their organs remain viable and can be transplanted due to mechanical ventilation and hemodynamic support [19].

Mitre et al. highlight another factor that may explain the reluctance of intensive care physicians in Romania to adopt the concept of brain death fully. They suggested that certain legislative frameworks may contribute to interpretative variability in brain death determination. However, the present study did not directly evaluate legal implementation processes [20]. Except in the context of organ transplantation, where legislation is clear and aligned with international norms, Law no. 95/2006 on Healthcare reform and Law no. 46/2003 on Patients’ Rights do not include the application of brain death criteria for other patients who develop an acute, unfavorable course toward death [21,22]. In these cases, a death diagnosis is based on cardiopulmonary arrest, which may translate into better knowledge and routine use of the latter. Our study brings this harsh reality to light. Most patients encountered in our participants’ daily activity are not potential organ donors, which translates into limited experience in applying brain death criteria. Only half of the surveyed ICU physicians know the brain death diagnostic criteria, with two-thirds agreeing with them. Approximately one-third of the participants contest or doubt the correctness of these criteria, associating them with a potential risk of false-positive results. Seventeen percent of surveyed physicians state that the brain death determination protocol is unclear, suggesting that a consistent educational process to improve knowledge and adoption of the brain death concept could contribute significantly to improved outcomes.

Our study is consistent with similar research stating that the concept of brain death has a major impact on ICU staff as well as on relatives, and establishing this diagnosis is a stressful experience [23,24]. Of the surveyed physicians, half state that the process of declaring brain death is an emotionally affecting experience. This may result in avoidance attitudes. Stavel’s quantitative research showed that only 16% of ICU staff would approach brain-dead patients for preservation aimed at organ donation, whereas 95% of participants surveyed by Boissy et al. stated they would participate in the donation process from donors after circulatory death [23,24].

Furthermore, our study shows that the emotional impact in the context of declaring death and interacting with relatives of the deceased is more pronounced among female physicians. Most of those who find the concept of brain death difficult for relatives to understand and who fear, to a large extent, a false-positive brain death diagnosis are women. Also, a significant proportion of those reporting major emotional impact after brain death diagnosis or considering the care of an organ donor in this condition, a stressful experience, are likewise women. This is nuanced by other research showing that emotional impact and professional burnout among female physicians may be up to 60% higher than among male physicians [25,26]. Potential causes include unique challenges many female physicians face, such as responsibility for family needs, reproductive role, infertility, inadequate compensation, lack of leadership opportunities, reduced mentorship and recognition, and lower salaries compared with their male colleagues. These findings could contribute to the development of more effective support and stress-management strategies for female physicians in such sensitive situations [25].

Despite this, the overall attitude toward organ donation observed in our study is favorable. Once brain death is established, most surveyed ICU physicians (84%) consider that organ donation should be the priority strategy for these patients. This highlights the conflict between healthcare workers’ intentions and the barriers created by a deficient system, represented by poor management, ambiguous legislation, and a lack of motivation, as 61.5% of responses indicate that organ procurement is not a priority in the ICUs where they work.

Regarding opinions about factors influencing donation rates in Romania, religion remains, in ICU physicians’ view, an extremely potent factor that continues to influence how Romanians relate to organ donation decisively. Although in one of our previous studies, conducted on the Romanian general population, we found that religiosity does not significantly impact positive attitudes toward donating one’s own organs [9], 80% of ICU physicians believe the influence of religious beliefs weighs heavily in donation decisions.

### 4.2. Communication with the Family of the Potential Brain-Dead Donors

Organ donation is a time- and resource-intensive process that imposes high demands on the medical team’s knowledge and involvement [27]. If organ donation is possible, the issue must be discussed with the patient’s close relatives [28]. Several factors are considered important for obtaining consent: effective communication with close relatives [29], competence and attitudes toward organ donation among medical staff [30,31], and relatives’ understanding of the consequences of brain functioning cessation [28,31].

An overwhelming majority of ICU physicians in our study consider that the decision regarding organ donation belongs to the family when the patient expressed no other option during their lifetime. The transition from the role of the patient’s treating physician to the role of ensuring optimal preparation for donation is compatible with this imperative only if it is carried out in accordance with the patient’s or their relatives’ wishes and does not unjustifiably influence end-of-life care [32]. This indicates that, from the ICU physicians’ perspective, informed consent is the only concept under which donation can be carried out in Romania, as suggested by Grigoraș and colleagues, who concluded that Romanian society is not prepared to accept the presumed consent legislation [33]. Nevertheless, it should be noted that even in societies that have adopted presumed consent, approximately half of ICU staff caring for potential donors consider that organ donation must occur with the family’s agreement [18].

Family refusal to accept organ donation is a complex phenomenon involving influenceable and non-influenceable factors, often in reciprocal interaction [34]. Sociodemographic factors, religious and cultural considerations, cause and circumstances of death, knowledge of brain death and donation concepts, trust in the healthcare system, awareness of the deceased’s wishes, satisfaction with the care provided, and the way consent is requested are recognized as key elements in the family’s decision [34,35,36,37]. Family members’ desire to preserve the body intact, lack of knowledge about the deceased’s wishes, and intrafamily disagreement were reported as the main causes of refusal [38,39,40,41]. Other predictors of family opposition include older age of potential donors, longer ICU stay, and lack of staff trained in organ procurement [38].

Similar to other research, our study shows that families’ inability to understand brain death remains an important cause of refusal of organ donation [38,42]. Eighty-seven percent of ICU physicians believe that relatives of Romanian patients are not familiar with the diagnosis of brain death, and 40% state that the concept of brain death is clear but difficult to explain to relatives. Moreover, 44% consider that expressing empathy toward relatives of brain-dead patients is more complicated than in other cases.

The concepts of brain death and interaction with relatives are considered particularly complex and sensitive. This stressful challenge places practitioners in a situation completely different from routine ICU situations [18]. While 63% of respondents reported that family discussions facilitate acceptance of the situation, 52% expressed concerns that such interactions could be perceived as inappropriate and potentially increase family distress. Although from the ICU physicians’ perspective, relatives are given sufficient time to consult among themselves, more than 60% believe these discussions may generate conflicts within families. This may be related to the emotional imbalance typical of the acute grief period, during which bereaved individuals need compassionate support, stabilization to return to physiological functional status (e.g., adjunctive medication for sleep), connection to social support systems, and encouragement of self-care activities [43]. It should be emphasized that bereaved persons have reported that donation helped them cope with sudden and unexpected loss, gave meaning to death, and brought comfort, considering that a part of the deceased continues to survive in the recipient’s body [38,44].

Other factors, such as the potential donor’s ICU stay, may translate into greater emotional exhaustion of relatives and a higher refusal rate. Additionally, longer ICU care could further amplify negative opinions regarding the conditions of treatment, different treatment protocols, clinical configurations, and personal experiences along the clinical pathway [38]. Another interesting aspect is provided by Piemonte and colleagues, who show that injury mechanism, pathology, etiology, and the cause of death may influence families’ decisions [38]. For example, families of patients with devastating brain injuries caused by trauma are more likely to consent to donation.

Previous research, such as the study by Roels et al., indicated that ICU team attitudes toward donation, acceptance of the brain death diagnosis, trust in the donation process, and communication skills were closely associated with relatives’ donation acceptance rates [30]. Other studies emphasized the importance of ICU staff qualification and experience in managing the procurement process and their direct consequences on consent rates. Alban et al. [45] described improved donor conversion rates in a U.S. trauma center after implementing specific educational programs, and Czerwinski reported the effectiveness of organ procurement training programs across all donation indicators [46].

Specific training rates among ICU staff for managing potential donor situations vary significantly, as mentioned in many studies [32,47,48]. While some studies report that only a small proportion of staff (3–22%) received specific training regarding brain death and approaching families about donation [18,49], in Romania, more than half of ICU staff state they are well prepared to manage situations where the family is willing to donate a brain-dead patient’s organs and to preserve the potential donor until donation. In a system where formal educational programs are lacking, the fact that a substantial proportion of ICU professionals possess competencies in caring for brain-dead patients may be explained by Romanian physicians’ motivation and capacity to stay one step ahead of the system, to exceed constraints through self-learning, and to develop professionally in an independent manner.

### 4.3. ICU Physicians’ Opinions Regarding the Transplant System and ICU Staff Involvement in Transplant Activity

The experience of medical staff working in intensive care units in Romania is relatively limited, with an average of eight brain-dead patients cared for annually by the participants in our study. Nevertheless, they are aware of their limitations, as only 60% consider themselves to possess adequate competencies in the management of brain-dead patients, in contrast to ICU physicians from other European countries, where 93.7% report being experienced in managing such cases [18].

Our study highlights the untapped potential to improve donation rates by motivating ICU personnel. More than 44% of respondents expressed a desire to become more involved in organ donation and transplantation activities. In comparison, 25.6% stated they would be willing to assume the role of Key Donation Person (KDP) or transplant coordinator. Of the total surveyed group, 63% indicated that medical staff could be motivated to increase their interest in identifying potential organ donors; however, only 38% considered financial incentives to be the most appropriate method. Opportunities for professional development through specialized continuing medical education courses, broader involvement in procurement and donation activities, psychological counseling, and recognition from authorities were among the motivational strategies reported by respondents.

A recurring theme in qualitative studies has been the identification of the potential for abuse and conflicts of interest between the priority of saving the donor’s life and achieving optimal outcomes in the organ donation process, which may generate substantial mistrust both from the public toward healthcare professionals and among healthcare professionals toward their colleagues [50,51]. Our study shows that more than 57% of respondents believe that, at present, there is no risk of abuse regarding organ procurement from brain-dead donors in Romania. Curley et al. reported that participants in their study feared that the image of the institution they worked for could be negatively affected if the public misinterpreted the goals of donation programs [50]. In the study conducted by Stavel and colleagues, the desire was expressed for the person responsible for end-of-life management to be different from the person involved in discussions with the family regarding organ donation, to avoid suspicions of potential abuse [24,52].

The systematic collection, storage, and comprehensive analysis of data in national registries constitute a major challenge; nevertheless, they provide substantial benefits, including ensuring equitable access to transplantation, generating a comprehensive overview of donation and transplantation activities, strengthening evidence-based clinical practice, enabling long-term monitoring of high-risk transplant recipients, and facilitating performance benchmarking across transplant centers [53]. Registries play a crucial role in establishing the evidence base for advancing transplant practice and ensuring transparency across the entire continuum of care, from organ donation to transplantation [53]. An overwhelming majority of the surveyed ICU physicians (87%) supported establishing a single national registry, including those willing to become organ donors. Furthermore, 77% of the participants supported the set-up of a donor card–based system as a feasible solution for maintaining a clear record of individuals who wish to become donors. Donor cards are considered a highly discreet and effective way to express the intention to donate organs after death, and many countries have adopted them as a central component of consent confirmation systems for organ donation. The decision to adopt such a system depends on multiple factors. Research indicates that individuals with lower levels of education, socioeconomic status, or poorer health status tend to be less enthusiastic about organ donation [54,55,56] and are less likely to sign a donor card or provide consent for donation [57,58,59,60]. Limited engagement with the topic, negative perceptions of organ donation, inadequate awareness of the demand for transplantable organs, and lack of trust in the healthcare system may collectively contribute to this phenomenon [61,62,63,64,65].

Further complicating the individual process of signing an organ donor card is the need to disclose this decision to family members. Open discussion regarding the intention to donate organs is essential. Family communication about organ donation is associated with higher levels of knowledge on organ donation and with the expectation that family members will respond positively to the idea of donation [66,67]. Smith et al. indicate that considering signing an organ donor card is associated with an increased willingness to discuss organ donation decisions with family members. Yet, only approximately half of registered donors reported communicating this decision to their families [66]. Furthermore, even when the deceased person’s consent to donation has been formally documented by signing a donor card, it has been reported that only a limited number of organ procurement organizations proceed with the donation if the family opposes the decision [66,68].

### 4.4. ICU Physicians’ Opinions from the Perspective of Potential Organ Donors or Family Members

It is widely recognized that medical education is associated with a favorable attitude toward organ donation and transplantation. Beginning in the first year of study, medical students demonstrate an increase in both knowledge levels and positive attitudes in this direction, which continue to intensify through the final year of training [69,70]. Among the ICU physicians included in our study, 66.7% expressed their intention to donate their own organs if they were declared brain dead. In comparison, 70% indicated their willingness to donate the organs and tissues of a family member under the same circumstances.

Although surveys indicate that the majority of physicians support organ donation, it remains unclear whether they are actually registered in donor databases to donate their own organs [71]. According to a Canadian study, physicians are more likely to register as organ donors than the general population, with younger age, female gender, residence in rural communities, and medical specialty (emergency medicine, internal medicine, pediatrics, or psychiatry) being significantly associated with higher donor registration rates [71]. Demonstrating that the majority of the medical community supports organ donation could positively influence the general population, serving as a model of engagement in this noble cause.

The observed gender differences may reflect broader patterns reported in the literature regarding emotional burden among physicians. However, given the exploratory design, this finding should be interpreted as hypothesis-generating rather than confirmatory.

### 4.5. Limitations

Several limitations should be acknowledged. The survey-based design relies on self-reports and may be subject to response bias. Although the questionnaire demonstrated good internal consistency, it was not externally validated. In addition, the sample size reflects an exploratory national assessment rather than a prevalence-estimating study. Nevertheless, the findings provide valuable system-level insights into professional attitudes, organizational barriers, and opportunities for improving organ donation pathways. As a survey-based study, these findings reflect real-world clinical experience and system-level constraints perceived by ICU physicians within the national transplant framework.

Also, the voluntary survey design introduces a potential selection bias, as participation may have been more likely among physicians with a pre-existing interest in organ donation or transplantation. The study is subject to response bias, including social desirability bias. Given the ethical sensitivity of brain death and organ donation, respondents may have provided answers aligned with perceived professional norms rather than reflecting personal uncertainty or institutional constraints. In addition, the cross-sectional nature of the survey limits causal inference. The study captures a unilateral professional perspective, as it includes only ICU physicians. The views of transplant coordinators, hospital administrators, policymakers, patients’ families, or the general population were not assessed. Therefore, the systemic conclusions should be interpreted within the boundaries of this professional viewpoint.

## 5. Conclusions

This nationwide survey-based quantitative study provides policy-relevant insights into intensive care physicians’ professional experiences, attitudes, and ethical perceptions regarding brain death and organ donation within the Romanian healthcare system.

This study represents the first nationwide policy-relevant assessment of ICU physicians’ perspectives on the Romanian transplant system, suggesting the presence of structural and educational gaps that may influence organ donation performance. Despite generally favorable professional attitudes toward organ donation, institutional prioritization remains inconsistent, while limited familiarity with brain death diagnostic criteria may be associated with variability in donor identification. Family consent remains the dominant determinant of donation, with inadequate public understanding of brain death and strong religious influences emerging as relevant contextual factors.

The findings suggest that further consideration of national standardization strategies may be warranted. Specifically, implementation of structured national training programs on brain death determination, standardized communication protocols for family discussions, formal integration of donor identification triggers into ICU clinical workflows, and mandatory notification pathways involving transplant coordinators may represent feasible clinical steps within the current system. Targeted workforce motivation strategies focused on professional development rather than financial incentives may further strengthen donor conversion rates. Addressing these systemic gaps may contribute to improved donor identification and coordination within the existing framework.

## Figures and Tables

**Table 1:** Descriptive statistical indicators of age (years).

N		117
Mean		41.03
Median		39.00
Std. Dev.		9.92
Variance		24.18
Skewness test		0.573
Std. error of Skewness		0.224
Minimum		26
Maximum		66
Percentile	25	32
	50	39
	75	47

## Data Availability

Data are contained within the article.
